# Supplementary material for: HIVprotI: an integrated web based platform for prediction and design of HIV proteins inhibitors
Source: J Cheminform. 2018 Mar 9;10:12. doi: 10.1186/s13321-018-0266-y (PMC5845081; doi:10.1186/s13321-018-0266-y)
Supplement: Supplementary file 2 — Additional file 2. Source code of HIVProtI web server. [file 13321_2018_266_MOESM2_ESM.zip › HIVprotI_Source-code/hivprotI/jsme/JSME_chemical_resolver_demo.html]

Your web browser must have JavaScript enabled
in order for this application to display correctly.

# JSME + Chemical Identifier Resolver demo

### Compound to name

The name of the molecule in the editor is fetched from the NCI web service (https://cactus.nci.nih.gov/chemical/structure) after the structure in the editor has changed.  

### Pasting any chemical structure representation

Paste operation accepts molecule name, Inchi, smiles ... and is using the same service.
  
Note: **the JQuery Ajax calls are not working on IE.**
  
  
Examples:
  
  
benazepril
  
c1ccccc1O
  
4-[(4-methylpiperazin-1-yl)methyl]-N-(4-methyl-3-{[4-(pyridin-3-yl)pyrimidin-2-yl]amino}phenyl)benzamide
  
50-78-2
  
PCZOHLXUXFIOCF-BXMDZJJMSA-N
